# Characterization of a stearoyl-acyl carrier protein desaturase gene family from chocolate tree, *Theobroma cacao* L

**DOI:** 10.3389/fpls.2015.00239

**Published:** 2015-04-14

**Authors:** Yufan Zhang, Siela N. Maximova, Mark J. Guiltinan

**Affiliations:** ^1^Huck Institutes of the Life Sciences, The Pennsylvania State University, University ParkPA, USA; ^2^Department of Plant Science, The Pennsylvania State University, University ParkPA, USA

**Keywords:** *Theobroma cacao*, stearoyl-ACP-desaturase, oleic acid, *ssi2*, cocoa butter, fatty acid composition

## Abstract

In plants, the conversion of stearoyl-ACP to oleoyol-ACP is catalyzed by a plastid-localized soluble stearoyl-acyl carrier protein (ACP) desaturase (SAD). The activity of SAD significantly impacts the ratio of saturated and unsaturated fatty acids, and is thus a major determinant of fatty acid composition. The cacao genome contains eight putative *SAD* isoforms with high amino acid sequence similarities and functional domain conservation with SAD genes from other species. Sequence variation in known functional domains between different *SAD* family members suggested that these eight *SAD* isoforms might have distinct functions in plant development, a hypothesis supported by their diverse expression patterns in various cacao tissues. Notably, *TcSAD1* is universally expressed across all the tissues, and its expression pattern in seeds is highly correlated with the dramatic change in fatty acid composition during seed maturation. Interestingly, *TcSAD3* and *TcSAD4* appear to be exclusively and highly expressed in flowers, functions of which remain unknown. To test the function of TcSAD1 *in vivo*, transgenic complementation of the Arabidopsis *ssi2* mutant was performed, demonstrating that TcSAD1 successfully rescued all AtSSI2 related phenotypes further supporting the functional orthology between these two genes. The identification of the major *SAD* gene responsible for cocoa butter biosynthesis provides new strategies for screening for novel genotypes with desirable fatty acid compositions, and for use in breeding programs to help pyramid genes for quality and other traits such as disease resistance.

## Introduction

*Theobroma cacao* L. (cacao) is an understory tropical tree domesticated in the Amazon basin and today widely cultivated in West Africa, Central and South America, and Southeast Asia (Argout et al., 2011). Cacao pods, containing around 40 seeds, are harvested approximate 20 weeks after pollination, and the seeds contain about 50% total lipids (cocoa butter), which provides a main raw ingredient for chocolate manufacturing as well as ingredients for pharmaceutical and cosmetic products (Liendo et al., 1997). Notably, cocoa butter composition consists of almost equal amount of palmitic acid (16:0), stearic acid (18:0), and oleic acid (18:1^n−9^), the composition of which determines its unique melting temperature very close to human body temperature, thus providing the smoothness and mouth feel of chocolate, as well as the creamy texture of cosmetics on skin (Liendo et al., 1997). Remarkably, during the 20 weeks of cacao seed development and maturation, dramatic transitions of fatty acid profiles occur within the only 20 days when a polyunsaturated fatty acid (linoleic acid (18:2^n−6^) and α-linolenic acid (18:3^n−3^))-rich membrane-like profile transitions to a final fatty acid content rich in saturated and monounsaturated-storage lipids (Patel et al., 1994). Moreover, as the dominant form of unsaturated fatty acid, 18:1^*n*−9^ gradually accumulates in developing cacao seeds, resulting in a significant impact on the physical properties of cocoa butter owing to its much lower melting temperature (16°C) compared to the other saturated fatty acids (16:0 – 62°C; 18:0 – 68°C) (Kachroo et al., 2007).

In plants, stearoyl-acyl carrier protein (ACP) desaturase (SAD, EC 1.14.99.6) is the nuclear-encoded, plastid-localized soluble desaturase that introduces the first Δ9 double bond into the saturated fatty acid resulting in the conversion of 18:0-ACP into 18:1^n−9^-ACP (Fox et al., 1993). As the dominant form of monounsaturated fatty acid exported from the plastid, 18:1^n−9^ can be further desaturated into polyunsaturated fatty acid derivatives, such as18:2^n−6^ and 18:3^n−3^, both of which serve as major components of cell membrane systems in the form of phospholipids (Ohlrogge and Browse, 1995). In this respect, given the fact that most plants lack other desaturases that utilize 18:0 as substrate, the activity of SAD is of particular interest because of its significant effects on the ratio of saturated fatty acids to unsaturated fatty acids (Lindqvist et al., 1996), and the effects on the fluidity and rigidity of membrane system and the relationship of this to the adaption of plants to various environmental conditions. For example, sunflower, flax, and castor grown in lower temperature conditions contained higher proportions of polyunsaturated fatty acid compared to those in high temperature growing regions (Green, 1986; Garces et al., 1992).

Given the functional importance of fatty acid saturation in plant development and industrial application, *SAD* genes from many plant species have been identified and characterized and a high correlations between the activities of SADs and levels of 18:0 and 18:1^n−9^ have been widely observed (Knutzon et al., 1992; Nishida et al., 1992; Slocombe et al., 1992; Gibson, 1993; Tong et al., 2006; Schluter et al., 2011; Shilman et al., 2011). For instance, a mutation of the Arabidopsis AtSSI2 gene, resulting in a single amino acid substitution resulted in an elevated level of 18:0 in Arabidopsis leaves (Lightner et al., 1994). Likewise, antisense-mediated reduction of SAD enzymatic activity in Brassica led to a dramatically increased level of 18:0 in the mature seed oil, from 2% up to 40% (Knutzon et al., 1992). Likewise, in soybean, a high 18:0 phenotype was strongly correlated with the activity of one of the SAD isozymes SACPD-C, an observation useful for screening of soybean varieties for unique seed oil profiles (Zhang et al., 2008).

In Arabidopsis, the family of acyl-ACP desaturases exhibits a high degree of amino acid sequence similarity (>70%) in spite of their minor functional differences (Kachroo et al., 2007). The three-dimensional crystal structure of homodimeric SAD protein from castor seed revealed that each SAD monomer comprises 11 conserved α-helices, which are crucial for ligand binding affinity, substrate chain-length selectivity, and double bond insertion position (Fox et al., 1993; Lindqvist et al., 1996; Cahoon et al., 1997). More impressively, replacements of several key amino acid residues in those conserved functional domains were sufficient to significantly alter the preferential mode of SAD. For example, replacement of five amino acids (T181A/F200A/N205S/T206L/A207G) of a castor Δ^9^ SAD resulted in a new specificity as a Δ^6^-16:0-ACP desaturase (Cahoon et al., 1997). Similarly, mutation of three amino acids (T117R/G188L/D280K) in castor Δ^9^ SAD enables it convert stearoyl-ACP to the allylic alcohol (*E*)-10-18:1-9-OH instead of 18:1^n−9^ (Whittle et al., 2008). Therefore, it is feasible to precisely manipulate activity of SAD taking advantage of the in-depth knowledge of its functional mechanism and advanced genetic approaches.

Interestingly, activities of SADs and levels of 18:1 are also involved in regulation of the defense response in many plant species via the fatty acid (FA)-derived signaling pathway (Kachroo and Kachroo, 2009). In Arabidopsis, decreased levels of 18:1^n−9^ in the *ssi2* mutant induced the SA mediated defense pathway, resulting in constitutive expression of *PR* genes, activation of the hypersensitive response and enhancement of broad-spectrum resistance to bacterial and oomycete pathogens (Shah et al., 2001; Kachroo et al., 2003, 2004). Similar defense responses were also observed in soybean and rice (Kachroo et al., 2008; Jiang et al., 2009), showing that silenced SAD activities were sufficient to induce the same type of defense-signaling pathway and enhance resistance to multiple pathogens. Together, it appears that 18:1 derived defense signaling pathway is conserved among many plant species. We recently demonstrated reduction of 18:1^n−9^ levels in cacao leaves, induced by application of exogenous glycerol, can induce a hypersensitive-like response and enhance the resistance against the oomycete pathogen *Phytophthora capsici* (Zhang et al., 2014b).

The cacao genome was previously shown to contain eight putative *SAD* genes (Argout et al., 2011). In this study, we examined the gene family in detail, and explored the expression patterns of each *SAD* gene in various cacao tissues. A single gene primarily involved in the synthesis of 18:1 pools in developing cacao seeds was identified and functionally characterized in Arabidopsis *ssi2* mutant. This information can be used to develop biomarkers for screening and breeding of new cacao varieties with novel fatty acid compositions of cocoa butter.

## Material and methods

### Gene identification, phylogenetic analysis, and protein prediction

*SAD* isoforms in *Theobroma cacao* were identified by blastp (Altschul et al., 1990) using full-length amino acid sequence of Arabidopsis AtSSI2 as the query (*E*-value cut-off 1e^−5^). Multiple protein sequence alignment was performed by MUSCLE (Edgar, 2004). The phylogenetic tree was constructed by MEGA4.1 using neighbor-joining algorithm with Poisson correction model and pairwise deletion (Tamura et al., 2007). Bootstrap values represent 1000 replicates. The phylogenetic tree was rooted using the amino acid sequence of PpSAD from *Physcomitrella patens*. Molecular mass and isoelectric point of SAD isozymes were predicted on ExPASy server (http://web.expasy.org/compute_pi/) (Gasteiger et al., 2005). Transient signal peptides were predicted using ChloroP 1.1 server (Emanuelsson et al., 1999) and TargetP 1.1 server (Emanuelsson et al., 2000).

Genbank accessions of the genes used in phylogenetic analysis:

**Table:** 

Gene	Accession	Gene	Accession	Gene	Accession
*TcSAD1*	KP704662	*AtSSI2*	At2g43710	*RcSAD1*	XP_002531889
*TcSAD2*	KP704663	*AtSAD1*	At5g16240	*RcSAD2*	XP_002526163
*TcSAD3*	KP704661	*AtSAD2*	At3g02610	*RcSAD3*	XP_002514780
*TcSAD4*	KP704664	*AtSAD3*	At5g16230	*RcSAD4*	XP_002514781
*TcSAD5*	KP704665	*AtSAD4*	At3g02620	*PpSAD*	XP_001772953
*TcSAD6*	KP704666	*AtSAD5*	At3g02630		
*TcSAD7*	KP704667	*AtSAD6*	At1g43800		
*TcSAD8*	KP704668				

### Fatty acid profiling by GC-MS

Plant tissue from glycerol treated and control leaves (four biological replicates) were ground in liquid nitrogen and fatty acid methyl esters (FAME) were prepared from each sample using approximately 30 mg of tissue per sample. Briefly, 1 ml of a MeOH/fuming HCl/Dichloromethane (10:1:1 v/v) solution was added to each tissue sample and incubated without shaking at 80°C for 2 h. Fatty acid methyl esters were re-extracted in 1 ml buffer H_2_O/Hexane/Dichloromethane (5/4/1, v/v) with vortexing for 1 min. The hexane (upper phase) was separated by centrifugation at 1500 g for 5 min, transferred to glass GC vials (Agilent) and evaporated to dryness under vacuum. The FAMEs were then dissolved in 500 μl hexane for GC-MS analysis. Pentadecanoic acid (C15:0) (Sigma, Cat. P6125) was used as the internal standard added prior to the extraction and methyl nonadecanoate (C19:0-methyl ester) (Sigma, Cat. N5377) was used as the spike control, added into the sample prior to the GC injection. Samples were analyzed on an Agilent 6890N gas chromatograph coupled to a Waters GCT time of flight mass spectrometer. Mass spectra were acquired in electron ionization mode (70 eV) from 45 to 500 Da at a rate of 1 scan/s. The samples were separated on an Omegawax^®^ 250 Capillary GC column (30 m × 0.25 mm 0.25 uM phase thickness, Sigma, Cat. 24136) using helium at a constant flow of 1.0 ml/min. The initial oven temperature was 100°C held for 1 min then increased at 15°C/min to a temperature of 150°C, and then increased at 4°C/min to a final temperature of 280°C. Samples (1 ul) were injected onto the column using a split/splitless injector maintained at 240°C with a split ratio of 50/1.

### RNA extraction and RT-qPCR analysis of gene expression

Plant tissues were first ground in liquid nitrogen. Three biological replicates for each tissue were subjected for the gene expression analysis. Total RNA was extracted using Plant RNA Purification Reagent (Life Technologies, Cat. 12322-012, following the manufactures instructions). The concentration of RNA was measured using a Nanodrop 2000c (Thermo Scientific). Five hundred nanograms of RNA was further treated with RQ1 RNase-free DNase (Promega, Cat. M6101) at 37°C for 30 min to remove potential genomic DNA contamination (following the manufacturer's protocol). The treated RNA was reverse-transcribed by M-MuLV Reverse Transcriptase (New England Biolabs) with oligo-dT_15_ primers to obtain cDNA. RT-qPCR was performed in total reaction volume of 10 μl containing 4 μl diluted-cDNA (1:50), 5 μl SYBR Green PCR Master Mix (Takara), 0.2 μl Rox, and 0.4 μl each 5 μM primers. Each reaction was performed in duplicates using Roche Applied Biosystem Step One Plus Realtime PCR System under the following program: 15 min at 94°C, 40 cycle of 15 s at 94°C, 20 s at 60°C, and 40 s at 72°C. The specificity of the primer pair was verified by PCR visualized on a 2% agarose gel and analysis of the qPCR dissociation curve. A tubulin gene (*Tc06g000360, TcTUB1, TcTUB1-5′*: GGAGGAGTCTCTATAAGCTTGCAGTTGG and *TcTUB1-3′*: ACATAAGCATAGCCAGCTAGAGCCAG) and a gene encoding an acyl-carrier protein (*Tc01g039970, TcACP1, TcACP1-5′*: GGAAAGCAAGGGTGTCTCGTTGAA and *TcACP1-3′*: GCGAGTTGAAATCTGCTGTTGTTTGG) were used as reference genes.

### Characterization of arabidopsis mutants and arabidopsis transformation

All Arabidopsis plants were grown in a Conviron growth chamber at 22°C with 16 h light/8 h dark cycle. Arabidopsis *ssi2* mutant was kindly provided by Dr. Kachroo (University of Kentucky), and homozygous *fab2* mutant (SALK_036854) was obtained from the Arabidopsis Biological Resource Center at Ohio State University (https://abrc.osu.edu/). The base pair mutation of *AtSSI2* in *ssi2* mutant was confirmed by sequencing the PCR product amplified by the following primers (LP1: TGAAGAAACCATTTACGCCAC; p3: CGTGTTGACATGAGGCAGATCG). The presence and homozygosity of T-DNA insertion in the *fab2* mutant line were confirmed by genotyping using the following primers (LB: CTTTGACGTTGGAGTCCAC; Up1F: TGAAACAGGTGCTAGTCCTACTTCA; Dn1R: CACCTGAAAGCCCGGTTAAGTC) (Schluter et al., 2011). Expression levels of *AtSSI2* in Col-0, *ssi2*, and *fab2* were examined by semi-quantitative RT-PCR using the following intron spanning primers (*AtSSI2-5′*: GGCCCCAAGGAGGTTGAGAG; *AtSSI2-3′*: ATCTGGAATGGATCCGCGGAC).

The full length coding sequence of *TcSAD1* was first amplified from leaf cDNA with the following primers (*TcSAD1-5′*-*XbaI*: GCTCTAGAATGGCTCTGAAATTGAATCCCAT and *TcSAD1-3′-XbaI*: TCTAGACTAGAGCTTCACTTCTCTATCAAAAATCCAAC). The resulting fragment was cloned into intermediate vector pE2113 using *XbaI*, driven under enhanced *E12-Ω CaMV 35S* promoter (Mitsuhara et al., 1996). The whole gene cassette was then cloned into the binary plasmid pCAMBIA-1300 (Hajdukiewicz et al., 1994) using *EcoRI* and *HindIII* sites, resulting in plasmid pGZ14.1021 (GenBank Accession KP100426) and was further transformed into *A. tumefaciens* strain *AGL1* by electroporation. Col-0, *ssi2*, and *fab2* Arabidopsis genotypes were transformed using the floral dip method (Clough and Bent, 1998). Transformants were selected on hygromycin B containing MS media (Harrison et al., 2006). Expression levels of *TcSAD1* in transgenic plants were confirmed by RT-PCR using the following primers: (*TcSAD1-RT-5′*: GCTCTGAAATTGAATCCCATCACTTCTCAA; *TcSAD1-RT-3′*: TGGCTCCAAAGAGGTTGAGAATGTC). Arabidopsis *Ubiquitin* (*AtUbi-5′*: ACCGGCAAGACCATCACTCT; *AtUbi-3′*: AGGCCTCAACTGGTTGCTGT) was used as a reference gene.

## Results and discussion

### The *T. cacao* contains eight highly conserved *SAD* isoforms in the genome

**Figure 1:**
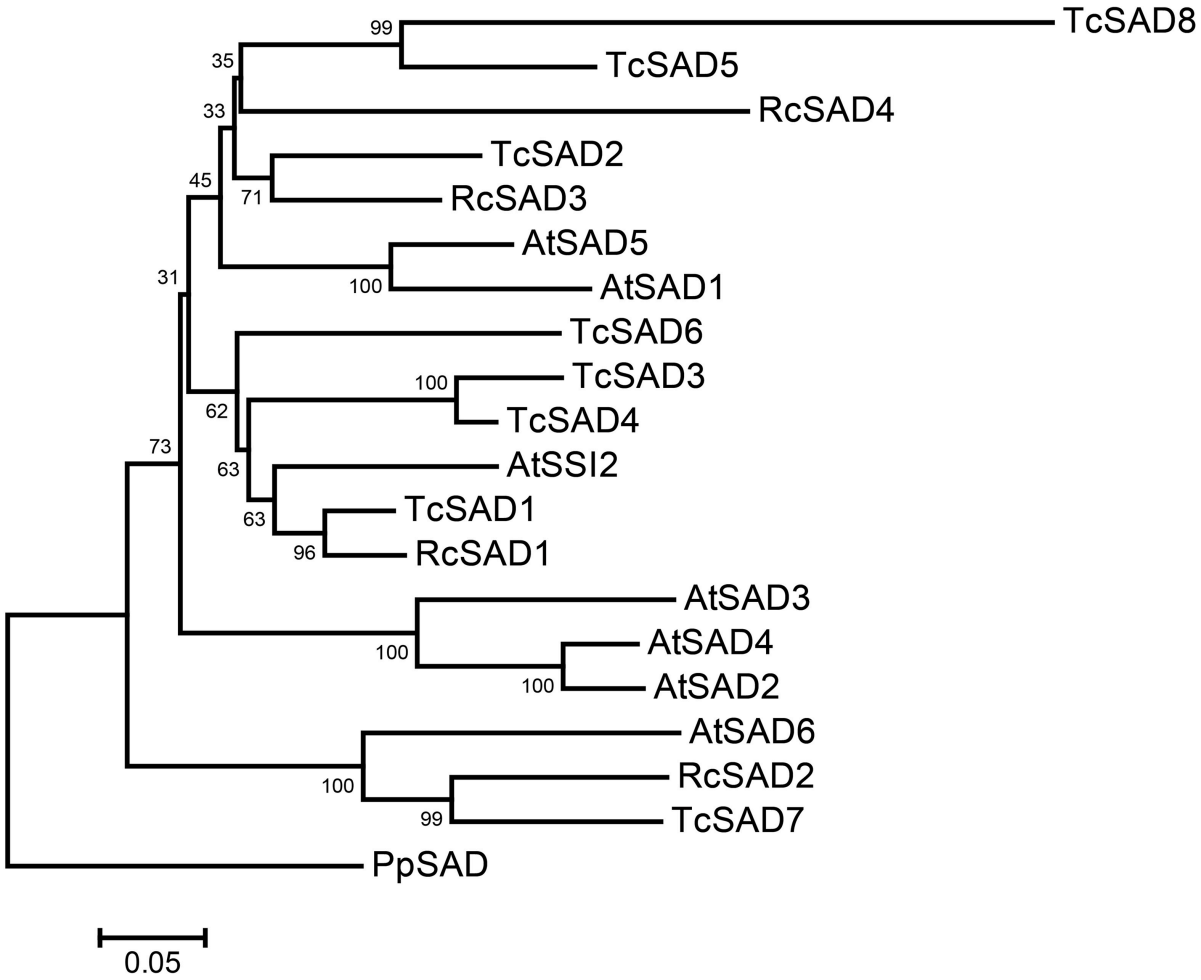
**Neighbor-joining bootstrap tree phylogeny based on full-length amino acid sequences of stearoyl-acyl carrier protein-desaturase (SAD) gene families in Arabidopsis (AtSAD), castor (RcSAD), and cacao (TcSAD)**. The scale bar represents 0.05 estimated substitutions per residue and values next to nodes indicated bootstrap values from 1000 replicates. The phylogenetic tree was rooted by using the amino acid sequence of PpSAD from *Physcomitrella patens*.

To identify the orthologous genes encoding *SAD*(s) in cacao genome, the full-length amino acid sequence of AtSSI2 (At2g43710) was blasted against the predicted cacao proteome of the Belizean Criollo genotype (B97-61/B2) (http://cocoagendb.cirad.fr/ Argout et al., 2011) using blastp algorithm with *E-value* cut-off 1e^−5^ (Altschul et al., 1990). Eight putative *SAD* genes were identified in cacao genome with reliable sequencing data and predicted gene structures (Table 1). Similar results were found with Blast analysis of the predicted cacao proteome of the genotype Matina1-6 (http://www.cacaogenomedb.org; Motamayor et al., 2013). The reciprocal best hits were further identified from both cacao genomes through blast and phylogenetic analysis (Supplemental File 1). Interestingly, of these eight *SAD* isoforms, four genes are located on chromosomes 4, and three of them (*Tc04g017510, Tc04g017520, Tc04g017540*) are clustered except for one predicted transposable element gene in between, which is similar to in the Arabidopsis genome where three out of seven *SAD* isoforms (*At3g02610, At3g02620*, and *At3g02630*) are located in tandem on chromosome 3 (Kachroo et al., 2007). Multiple amino acid sequence comparison and phylogenetic analysis including SAD isoforms from cacao, castor, and Arabidopsis revealed that Tc04g017510 (designated as TcSAD1) is closely clustered with RcSAD1 and AtSSI2 (Figure 1), both of which have significant impacts on oleic acid contents and fatty acid profiles in Arabidopsis (Lightner et al., 1994) and castor (Lindqvist et al., 1996), respectively, suggesting a potential significant role of TcSAD1 in cacao seed oil biosynthesis. Moreover, Tc05g012840 (designated as TcSAD2), Tc04g017520 (designated as TcSAD3), and Tc04g017540 (designated as TcSAD4) also exhibit high sequence similarities to RcSAD1, implying that they may also contribute to the activity of SAD in cacao.

**Table 1:** **Blastp result using AtSSI2 (At2g43710) as the query**.

**Query**	**Locus ID**		**Designation**	**Expect**	**% Identity**	**Query Coverage**	**Hit Coverage**
	**Criollo**	**Matina1-6**					
AtSSI2	Tc04g017510	Thecc1EG019858	TcSAD1	0	80.05	100	99.75
	Tc05g012840	Thecc1EG023383	TcSAD2	1E-177	80.91	87.53	89.09
	Tc04g017520	Thecc1EG019859	TcSAD3	1E-176	80.97	87.78	88.66
	Tc04g017540	Thecc1EG019861	TcSAD4	1E-174	80.86	87.28	95.63
	Tc04g005590	Thecc1EG017405	TcSAD5	1E-166	76.49	87.53	89.37
	Tc09g024040	Thecc1EG040800	TcSAD6	1E-144	72.7	84.04	97.88
	Tc08g012550	Thecc1EG035438	TcSAD7	1E-143	70.12	83.29	86.89
	Tc01g009910	Thecc1EG001225	TcSAD8	2E-71	42.98	86.53	89.97

The metabolic pathways in which SADs participate in are well-known to occur in the lumen of plastids (Cahoon et al., 1997). Thus, we performed bioinformatics analysis of transit peptides (TP) in the N-terminal sequences of the predicted cacao SAD proteins using the TargetP (Emanuelsson et al., 2000) and ChloroP (Emanuelsson et al., 1999) algorithms. AtSSI2 (At2g43710) in Arabidopsis and RcSAD1 (XP_002531889) in castor were also included in this analysis since the structures and the functions of these two proteins have been well-studied (Lindqvist et al., 1996; Cahoon et al., 1997; Kachroo et al., 2007; Whittle et al., 2008). High-confidence chloroplast transient peptides were successfully detected in AtSSI2 and RcSAD1, consistent with previous analysis (Kachroo et al., 2007; Schluter et al., 2011). Of eight cacao SAD isoforms, chloroplast transient peptides were identified in TcSAD1, TcSAD2, TcSAD5, and TcSAD7. A mitochondrial targeting peptide was predicted in TcSAD3 and a signal peptide related with the secretory pathway was detected in TcSAD4, suggesting that these isoforms may exhibit distinct functions in mitochondria. No transient peptides were detected in TcSAD6 and TcSAD8 (Table 2).

**Table 2:** **Predicted protein characteristics of cacao, Arabidopsis, and castor SAD proteins**.

**Designation**	**Locus ID**	**pI (isoelectric point)**	**MW (kDa)**	**Length (a.a)**	**Score**	**TP-length (a.a)**	**Predicted localization**
TcSAD1	Tc04g017510	6.05	45.39	396	0.564	33	C
TcSAD2	Tc05g012840	6.24	44.83	393	0.536	27	C
TcSAD3	Tc04g017520	5.87	45.31	396	0.508	33	M
TcSAD4	Tc04g017540	5.33	41.86	365	0.43	26	S
TcSAD5	Tc04g005590	6.51	44.76	394	0.546	28	C
TcSAD6	Tc09g024040	5.49	37.94	329	0.434	21	–
TcSAD7	Tc08g012550	6.39	44.08	388	0.538	38	C
TcSAD8	Tc01g009910	5.88	33.91	298	0.448	21	–
AtSSI2	At2g43710	6.61	45.65	401	0.587	35	C
RcSAD1	XP_002531889	6.19	45.37	396	0.557	33	C

*Transit peptide (TP) length and subcellular localization are indicated (C, Chloroplast; M, Mictochondrial; S, Secretory pathway)*.

### SAD isozymes in *T. cacao* share conserved overall protein structures with noteworthy differences

**Figure 2:**
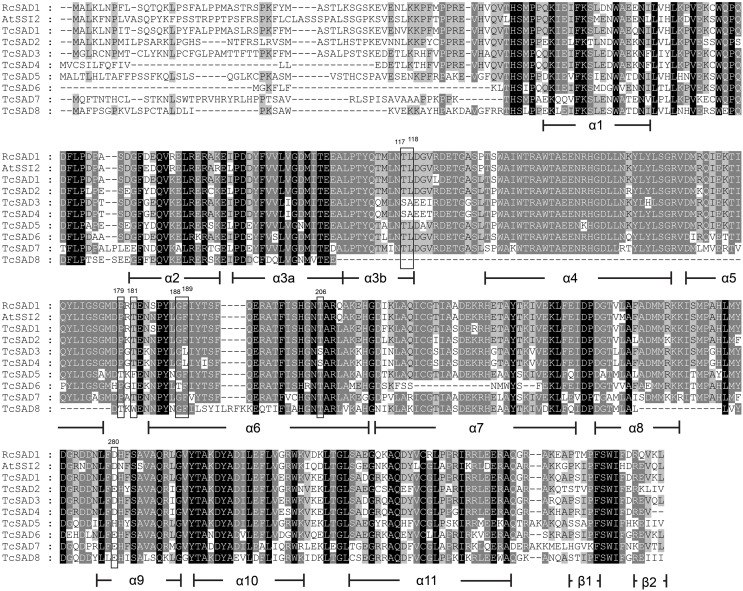
**Multiple amino acid sequence alignment of TcSADs from cacao with SAD from castor bean (RcSAD1) and Arabidopsis (AtSSI2)**. Secondary protein structures of SAD were compared, deduced, and annotated according to the crystal structure of RcSAD1. Shadings were performed by Genedoc software (Nicholas et al., 1997) in a conservation mode. Residues in black represent 100% conservation of all sequences; residues in dark gray represent >75% identity of all sequences; residues in light gray >50% identity of all sequences. Functional determinative amino acids were marked in rectangles and numbered according to the start codon of RcSAD1.

The three-dimensional protein structure of RcSAD1 from castor seed has been extensively studied in detail as a model of Δ^9^ desaturase in plants (Schneider et al., 1992; Fox et al., 1993; Lindqvist et al., 1996). To provide further insights into potential catalytic activities of SAD isozymes in *T. cacao*, a multiple amino acid sequence alignment was conducted including RcSAD1, AtSSI2, and eight cacao SADs, and the determinative amino acid residues that are highly associated with substrate binding activities and chain length specificities of the SADs were evaluated (Figure 2). As mentioned above, a high degree of variation was observed within putative transient signal peptides in N-terminal sequences. Consistent with crystal structure of RcSAD1, cacao SADs also consist of 11 highly conserved α-helices, except that putative TcSAD8 lacks of the region from α3b- to α5-helices, which might greatly compromise its normal functionality. Notably, a further detailed investigation of the determinative residues revealed that several prominent divergences exist within cacao SADs, implying that they might have distinct substrate preferences. For example, amino acid residues at positions of 117, 118, 189, and 206 (positions assigned according to castor RcSAD1 amino acid sequence, indicated in Figure 2) were reported to be crucial for the function of RcSAD1 as a Δ^9^-18:0-ACP desaturase, and replacements of these residues converted RcSAD1 into an enzyme that either functioned as a Δ^6^-16:0-ACP desaturase or converted 18:0-ACP into the allylic alcohol *trans*-isomer (E)-10-18:1-9-OH (Cahoon et al., 1997; Whittle et al., 2008). In this respect, TcSAD3 and TcSAD4 share the same amino acid residues at those positions which differ from RcSAD1, AtSSI2, and other cacao SADs, suggesting that TcSAD3 and TcSAD4 may exert different functions from the other cacao SADs. Likewise, at positions of 179 and 181, which are crucial for the substrate specificity of RcSAD1 (Cahoon et al., 1997), TcSAD5, TcSAD6, and TcSAD8 contain unique varied amino acid residues compared to RcSAD1, AtSSI2, and the other cacao SADs.

### Identification and validation of housekeeping genes across *T. cacao* tissues

**Figure 3:**
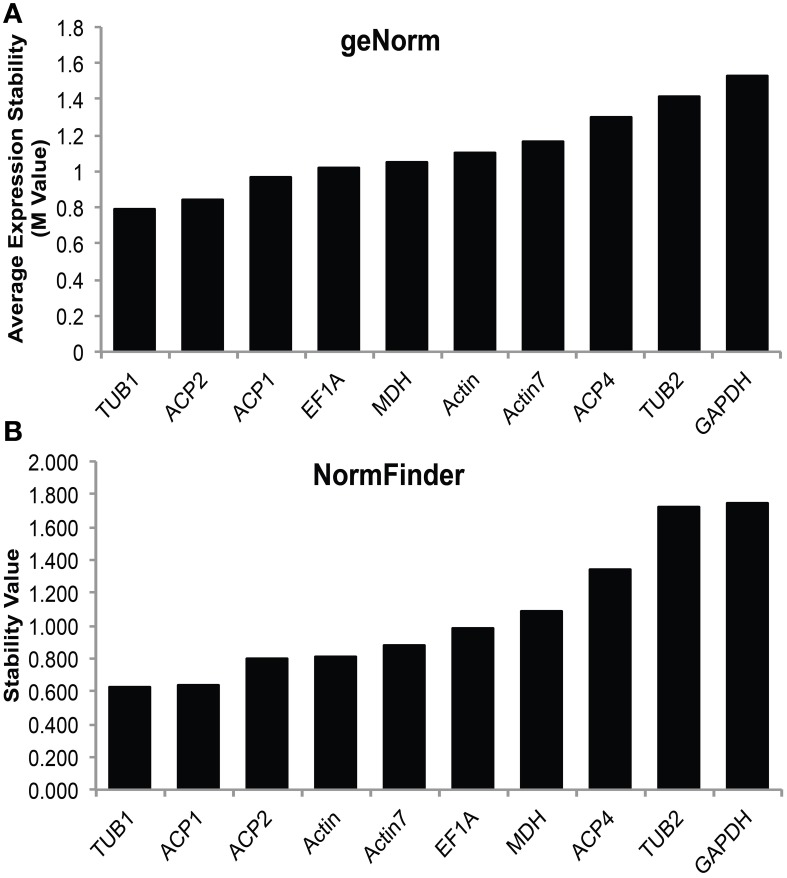
**Stability values of candidate reference genes**. **(A)** Average expression stability *M*-values of expression levels of candidate reference genes in various cacao tissues calculated by geNorm software. **(B)** Stability value of candidate reference genes in various cacao tissues calculated by NormFinder software. Tissues include: leaves stage A, C, and E [early, mid, and late development, see (LA, LC, LE), developing flowers (UF) and mature flowers (OF), root (Rt), and zygotic cotyledons from seeds collected at 14, 16, 18, and 20 weeks after pollination (14, 16, 18, and 20 Cot)].

In order to accurately characterize the expression pattern of SAD isoforms, suitable reference genes for qRT-PCR normalization were first identified and validated in various cacao tissues including: leaves at developmental stages A, C, and E (defined in Mejia et al., 2012), unopened flowers, open flowers, roots, and zygotic seeds at 14, 16, 18, and 20 weeks after pollination (WAP). Ten candidate genes primarily involved in cellular structures and central metabolisms were selected and evaluated including: tubulin (*TUB*), actin (*ACT*), acyl carrier protein (*ACP*), elongation factor (*EF1α*), glyceraldehyde-3-phosphate dehydrogenase (*GAPDH*), and malate dehydrogenase (*MDH*). All the genes were successfully amplified in all the tested cacao tissues using gene specific primers that preferentially bind near the 3′ end of the cDNA (Supplemental Table 1). The specificities of the primers were confirmed by melting curve analysis (Supplemental File 2). The reference gene stability analysis was conducted applying two statistical approaches: geNorm (Vandesompele et al., 2002), which calculates *M*-values to suggest the mean expression stability of given genes, and NormFinder (Andersen et al., 2004), which estimates not only the overall variation of candidate reference genes but also the variation within biological sample subgroups and experimental conditions. According to both geNorm and NormFinder algorithms, *TUB1* (*Tc06g000360*) has the lowest average expression stability values (Figures 3A,B), suggesting *TUB1* is the most stable gene in the tested gene set across all these cacao tissues. Therefore, *TUB1* was selected as the reference gene to characterize the gene expression pattern of *SAD* isoforms in various cacao tissues. Additionally, given central roles of ACPs in transporting and stabilizing the growing fatty acid carbon chains throughout the fatty acid biosynthetic pathway (Crosby and Crump, 2012), *ACP1* was selected as the reference gene to normalize gene expression level during cacao seed development since *ACP1* was also recognized as the second most stable reference gene from NormFinder analysis (Figure 3B).

### Tissue specific expression pattern of *SAD* isoforms in *T. cacao*

**Figure 4:**
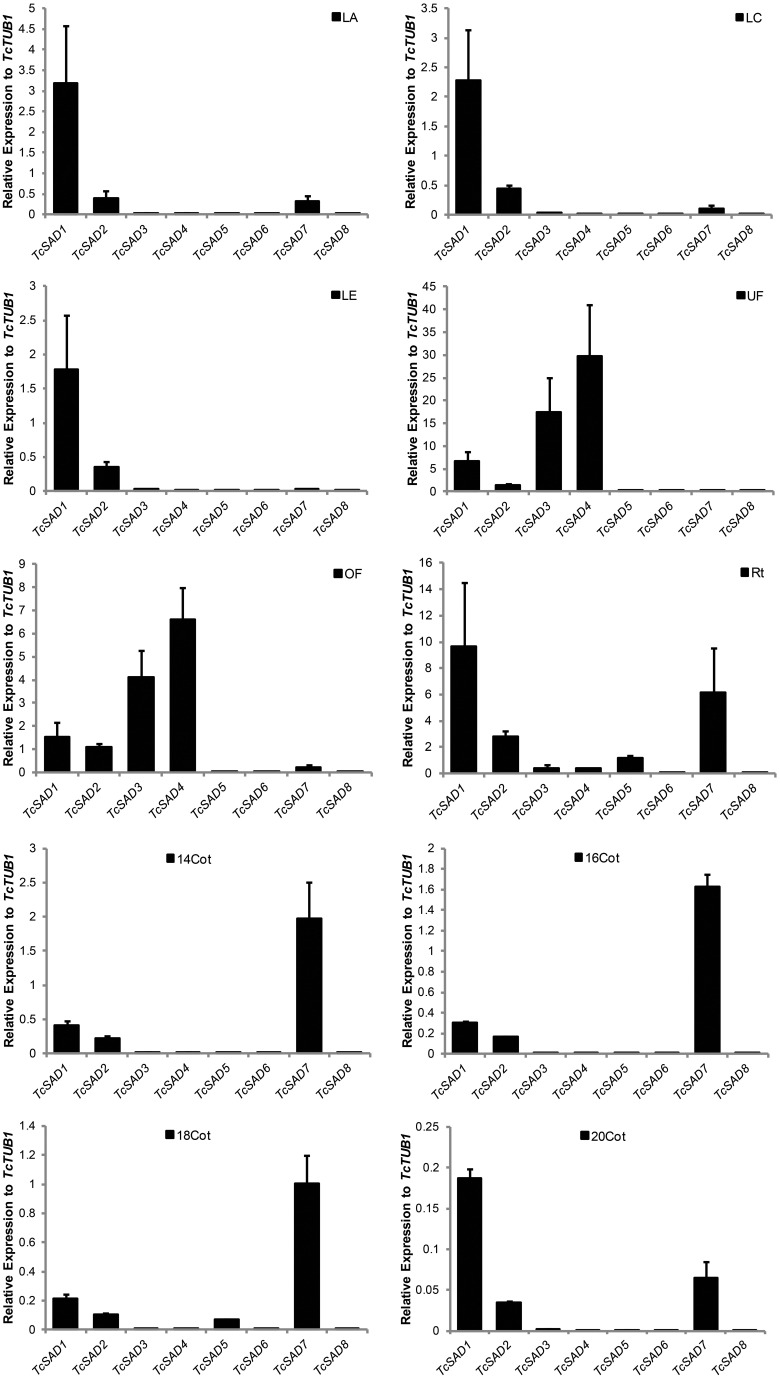
***TcSAD* expression patterns in various cacao tissues**. Bar charts illustrate the relative expression levels of *TcSAD* isoforms in various cacao tissues normalized to that of *TcTUB1* measured by qRT-PCR. The same set of cacao tissues as indicated in Figure 3 was included in this analysis. Three biological samples for each tissue were included in the analysis.

To further characterize functions of the cacao *SAD* family, the expression profiles were examined by qRT-PCR in various cacao tissues including: leaves at developmental stages A, C, and E (defined in Mejia et al., 2012), unopened flowers, open flowers, roots, and zygotic seeds at 14, 16, 18, and 20 WAP. Since *SAD* isoforms are highly conserved in their functional domains (α-helices and β-sheets), isoform specific qRT-PCR primers were designed to preferably target at predicted 5′ and 3′ untranslated regions, and signal transient peptide regions, all of which exhibited low sequence similarities among *SAD* isoforms in cacao (Supplemental Table 2). The specificities of each set of primers were determined by examining amplification products on the agarose gel and further confirmed by melting point analysis (Supplemental File 3). Expression levels of *SAD* isoforms were normalized to *TcTUB1* (Table 3). As shown in Figure 4, transcripts of *TcSAD1* and *TcSAD2* were detected in all the examined tissues, in which the expression levels of *TcSAD1* were higher than those of *TcSAD2*. Interestingly, *TcSAD3* and *TcSAD4* were predominantly expressed in unopened and open flowers, where the expression levels of *TcSAD3* and *TcSAD4* were significantly higher than any other *SAD* isoforms; however, the transcripts of both were barely detectable in all the other tissues, indicating that they are flower specific genes. The expression of *TcSAD5* was primarily detected in roots; however, at levels lower than those of *TcSAD1, TcSAD2*, and *TcSAD7* in roots. *TcSAD7* constitutively expresses in all examined tissues with higher expression in roots and zygotic seeds at all developmental stage (Figure 4). Overall, on the basis of gene expression data, both *TcSAD1* and *TcSAD7* contribute to the major proportions to the total *SAD* transcripts in developing zygotic embryos, with *TcSAD7* being higher expressed over all the stages except in zygotic seeds at 20 WAP, implying that the activities of both TcSAD1 and TcSAD7 are possibly involved in the synthesis and accumulation of 18:1 in cacao embryos (Figure 4). Notably, the transcripts of *TcSAD6* and *TcSAD8* were barely detected in any of the tissues analyzed, suggesting that they might be pseudogenes or expressed in a tissue, stage of development or induction condition not tested.

**Table 3:** **Summary of relative expression levels of *TcSAD* isoforms in various cacao tissues**.

	**Expression pattern**	**Predominance**	**LA**	**LC**	**LE**	**UF**	**OF**	**Rt**	**14 Cot**	**16 Cot**	**18 Cot**	**20 Cot**
TcSAD1	Constitutive	Lower in embryos	3.1736±1.4003	2.2747±0.8641	1.7835±0.7933	6.6441±2.1348	1.5259±0.6346	9.633±4.888	0.4123±0.0608	0.2997±0.0191	0.2159±0.0294	0.187±0.0113
TcSAD2	Constitutive	Low	0.397±0.1732	0.449±0.0554	0.3587±0.0806	1.3392±0.465	1.1114±0.1451	2.8314±0.3767	0.2243±0.0278	0.1643±0.0041	0.1039±0.0098	0.0345±0.0017
TcSAD3	Flowers	~30% of total expression	0.0328±0.0112	0.0328±0.0096	0.0274±0.0138	17.535±7.4756	4.1346±1.1218	0.4207±0.2133	0.0172±0.0023	0.0084±0.0023	0.0042±0.0007	0.0018±0.0006
TcSAD4	Flowers	~40% of total expression	0.0309±0.0102	0.0231±0.0007	0.0174±0.0016	29.6626±11.4378	6.6223±1.3677	0.351±0.0339	0.0182±0.0015	0.0085±0.0017	0.0027±0.0007	0.0005±0.0001
TcSAD5	Root & Embryos	Low	0.005±0.001	0.0004±0.0002	0±0	0.0434±0.0164	0.0131±0.0029	1.1332±0.1956	0.0015±0.0004	0.016±0.0027	0.0711±0.0026	0.0009±0.0004
TcSAD6	–		0.0181±0.0012	0.0019±0.0005	0.0015±0.0003	0.0064±0.0029	0.0032±0.0011	0.0042±0.0007	0.0026±0.0017	0.0008±0.0003	0.0003±0.0002	0.0003±0.0001
TcSAD7	Constitutive	High in roots & embryos	0.3271±0.1328	0.1102±0.0555	0.0255±0.019	0.1993±0.0737	0.2312±0.0889	6.1258±3.4435	1.9744±0.5346	1.6229±0.1256	1.0066±0.1921	0.0656±0.0191
TcSAD8	–		0±0	0±0	0±0	0.0001±0.0001	0±0	0.0001±0.0001	0.0013±0.0018	0.0001±0.0001	0±0	0±0

Profoundly, *TcSAD3* and *TcSAD4* appear to be exclusively and highly expressed in floral tissues. These two proteins are unique within the cacao *SAD* gene family in containing predicted mictochondrial targeting and secretory peptides respectively (Table 2). Interestingly, a previous study in orchid revealed that the activities of SAD isoforms were involved in the biosynthesis of alkene species with different double bond positions, which result in the major odor differences among orchid species attracting differential pollinators (Schluter et al., 2011). Moreover, a more recent study in tobacco showed that the normal function of one isoform of stearoyl-ACP desaturase was critical to maintain membrane lipid composition during ovule development and thus affected female fertility (Zhang et al., 2014a). Therefore, even though functions of TcSAD3 and TcSAD4 in cacao floral tissues remain largely unknown, it is reasonable to speculate that their activity might be associated with the synthesis of fatty acid derived metabolites that are vital to the pollination process and the development of cacao reproductive tissues.

### Oleic acid accumulation and *SAD* isoform expression during cacao seed development

**Figure 5:**
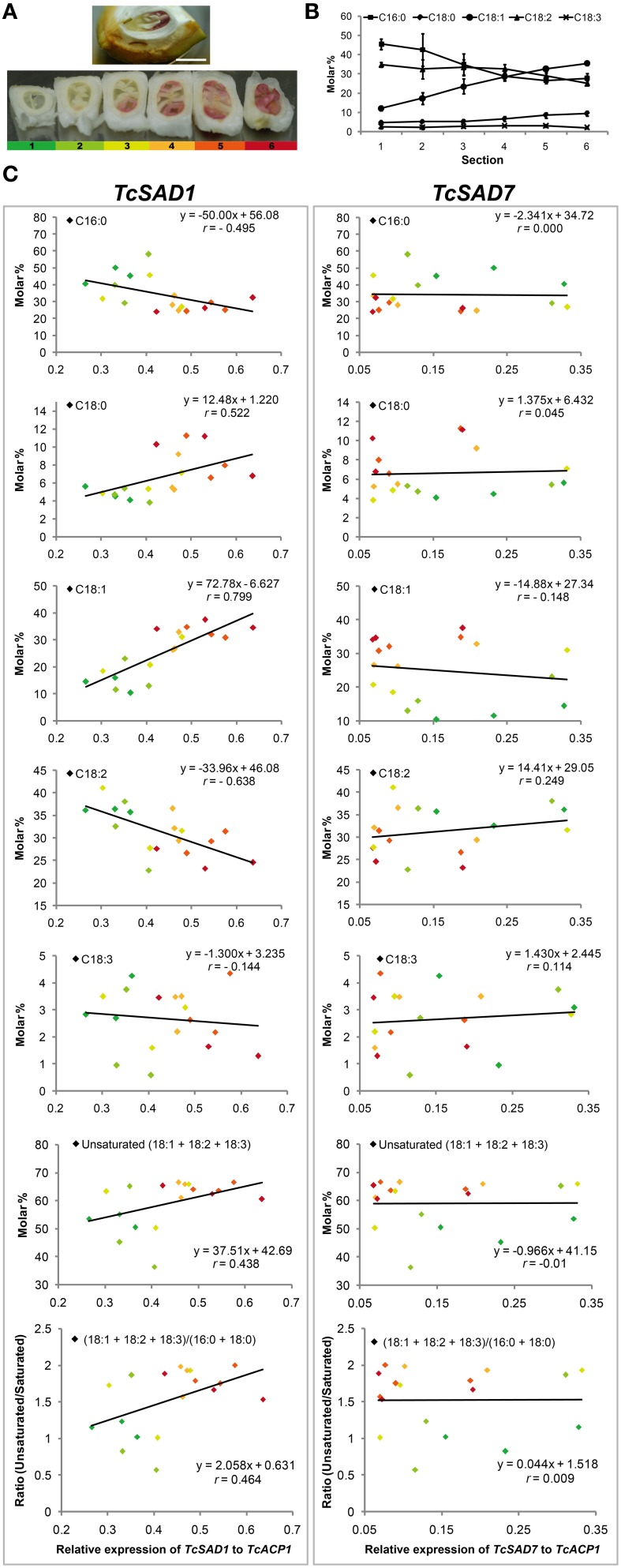
***TcSAD* relative expression and fatty acid composition in maturing cacao seeds. (A)** (Upper) Mid-stage cacao seed sliced longitudinally, within ovule and exocarp tissue (pod shell) 12 weeks after pollination. The embryo root axial meristem is to the right, expanding cotyledonary tissues to the left. (Lower) Six radial serial thick sections dissected from a single cacao seed oriented as in the top image, then rotated 90 degrees to display the cross sections. Each section is sequentially numbered from left to right and color coded by green, light green, yellow, orange, light red, red, respectively, from youngest cotyledonary end (left) to the most mature axial end (right). Scale bar = 1 cm. (**B**) Molar percentages of fatty acid compositions in each seed section. (*n* = 3, mean ± SE). **(C)** The correlation of *TcSAD1* and *TcSAD7* gene expression and molar percentage of fatty acid composition in various cross sections of developing cacao seeds. Left column, *TcSAD1*, right column, *TcSAD7*. Color-coded data points represent different seed sections as labeled in **(A)**. Three biological replicates from each seed section were analyzed. Each independent measurement is shown as a separate data point. Correlation coefficient *r* was calculated based on the linear regression model by Minitab (Ryan et al., 2004).

Notably, the maturation of cacao seeds initiates from the axial ends and gradually toward the cotyledonary ends. Seeds during this rapid transition are ideal to evaluate the correlation between oleic acid contents and the activities of *TcSAD*s since biological variations are greatly minimized. To explore the role of each TcSAD isoform in seed lipid biosynthesis, maturing cacao seeds were isolated from fruit at 12 WAP (Figure 5A), and the entire cacao seed was transversely dissected into six segments along the developmental gradient, with the least developed segment (segment 1, color coded as dark green, Figure 5A) at the cotyledonary end and the most developed segment (segment 6, color coded as dark red, Figure 5A) at the axial end. The analysis of fatty acid composition in each segment indicated that the percentage of 18:1^n−9^ gradually increased from 12% in segment 1 to 35% in segment 6 (Figure 5B), which is consistent with previous reports of fatty acid composition changes during cacao seed development over time (Patel et al., 1994). Thereafter, the expression of two major *TcSAD* isoforms in seeds (*TcSAD1* and *TcSAD7*, Figure 4) was examined in each segment by qRT-PCR, and the correlation between the expression levels of *TcSAD*s and fatty acid composition was evaluated by linear regression (Figure 5C). Expression levels of *SAD* isoforms were normalized to *TcACP1*. In general, the levels of *SAD* gene expression shown in Figure 5C suggested that *TcSAD1* expression (left panel) was highly correlated with gradual alterations of fatty acid composition during development. However, expression of *TcSAD7* did not correlate well with fatty acid composition changes (right panel). Specifically, *TcSAD1* expression was positively correlated with the molar percentage of 18:1^n−9^ (*r* = 0.799) and 18:0 (*r* = 0.522), and negatively correlated with the molar percentage of 18:2^n−6^ (*r* = −0.638); however, poor correlations were observed between the expression of *TcSAD7* and these fatty acid species. Additionally, the expression of *TcSAD1* was also positively correlated with the level of total molar percentage of unsaturated fatty acids (18:1^n−9^ + 18:2^n−6^ + 18:3^n−3^) (*r* = 0.438) and the ratio of unsaturated fatty acid (as above) over saturated fatty acid (16:0 + 18:0) (*r* = 0.464). Again, the expression of *TcSAD7* was poorly correlated with these two aspects.

Taken together, the evidence suggests that TcSAD1 is the dominant isoform for the synthesis of seed storage fatty acids in cacao since the increasing expression levels of *TcSAD1* were highly correlated with the gradual changes of fatty acid compositions. It further suggests that TcSAD7 may play a minor role in this process and could play additional roles in seed development unrelated to storage lipid accumulation. Regarding this, a recent characterization of *AtSAD6*, a *TcSAD7* homolog in Arabidopsis, revealed that AtSAD6 may be involved in the fatty acid desaturation and lipid metabolism in crown galls under hypoxia and drought stress conditions (Klinkenberg et al., 2014).

### TcSAD1 is functional equivalent to AtSSI2

**Figure 6:**
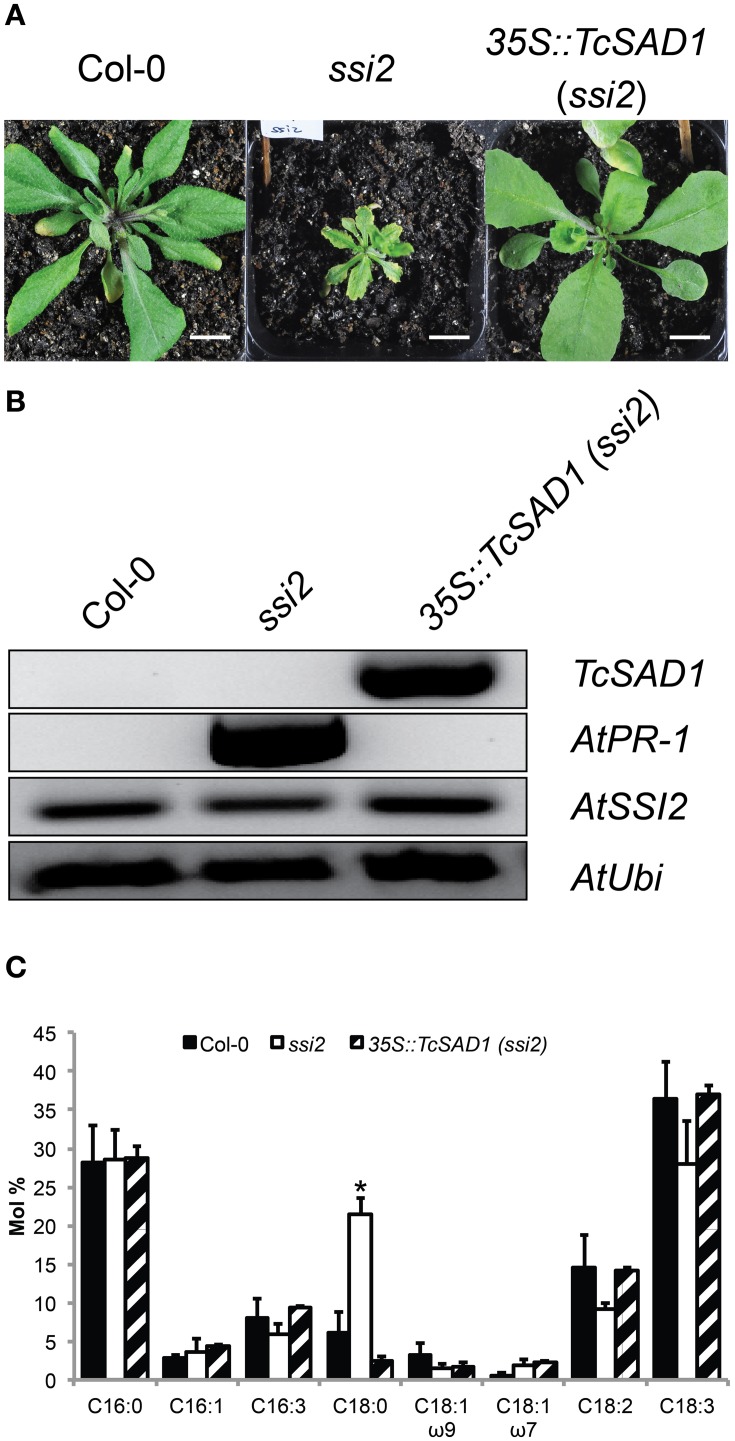
**Transgenic complementation of the Arabidopsis *ssi2* mutation with the cacao TcSAD1 gene**. Comparison of morphological, molecular, and fatty acid composition phenotypes of *ssi2* and *35S::TcSAD1* (*ssi2*) plants. **(A)** Morphological comparison of wild-type Col-0, *ssi2*, and *35S::TcSAD1* (*ssi2*) at 4-week-old plants. Overexpression of *TcSAD1* in Arabidopsis *ssi2* successfully rescued dwarf phenotype and eliminated hypersensitive responses. Scale bar = 1 cm. **(B)** Overexpression of *TcSAD1* eliminated constitutive overexpression of *AtPR1* (At2g14610) in *ssi2*, but did not affect the level of *AtSSI2*. Arabidopsis *Ubiquitin* was used as the reference gene in RT-PCR analysis. **(C)** Fatty acid compositions of wild-type Col-0, *ssi2*, and *35S::TcSAD1* (*ssi2*) (*n* = 6 for Col-0 and *ssi2, n* = 2 for *35S::TcSAD1*, mean ± SD).

To further investigate *in vivo* TcSAD1 and TcSAD7 protein functions, *TcSAD1* and *TcSAD7* were overexpressed in Arabidopsis *ssi2* mutant background, driven by *Cauliflower mosaic virus* 35S promoter. Owing to the poor transformation rate and mortality rate of the *ssi2* mutant (Schluter et al., 2011), only six transgenic lines overexpressing *TcSAD1* were identified; however, no transgenic plant overexpressing *TcSAD7* was obtained from three Arabidopsis transformation attempts. Notably, two out of six *35S::TcSAD1* (*ssi2*) transgenic lines were morphologically indistinguishable from wild type Col-0 plants (Figure 6A), suggesting that the function of TcSAD1 successfully rescued the dwarf phenotype of *ssi2* mutant. RT-PCR analysis confirmed that transcripts of *TcSAD1* were only detected in the transgenic lines, but not in wild type or *ssi2* mutant plants (Figure 6B). Fatty acid composition analysis on wild type Col-0, *ssi2* mutants and the *35S::TcSAD1* (*ssi2*) transgenic lines revealed that overexpression of *TcSAD1* restored the wild type-like level of 18:0 (Figure 6C). Another dramatic phenotype of the Arabidopsis *ssi2* is the constitutive expression of *PR* genes and spontaneous cell death (Shah et al., 2001). In this respect, *35S::TcSAD1* (*ssi2*) plants exhibited no visible cell death on the leaves and contained only basal level of *PR-1* expression (Figure 6B). Taken together, we concluded that the overexpression of *TcSAD1* was sufficient to functional complement the reduced activity of SSI2 in the *ssi2* Arabidopsis mutant, demonstrating that TcSAD1 is a functional ortholog of AtSSI2.

## Conclusion

In conclusion, the eight *TcSAD* isoforms in cacao genome share high amino acid sequence conservation but they also have specific differences in key determinative amino acid residues and distinct tissue specific expression patterns. Among them, *TcSAD1* is phylogentically most closely clustered with *RcSAD1* and *AtSAD1*, and is functional equivalent to *AtSAD1*. Notably, the expression level of *TcSAD1* was also highly positively correlated with the level of 18:1^n−9^ and therefore affected final fatty acid profiles in maturing cacao seeds, which makes it as an ideal biomarker to screen for desirable fatty acid compositions of cocoa butter. *TcSAD1* successfully complimented the Arabidopsis *ssi2* mutant. Together, our results strongly support the conclusion that TcSAD1 is the functional ortholog of AtSSI2, and plays a major role in determining the fatty acid composition of cacao seeds.

## Authors contributions

YZ conceived and performed all the experiments and drafted the manuscript. SM was involved in designing and directing the experiments, and revising the manuscript. MG contributed to the conception of the study, gave advice on experiments, and finalized the manuscript.

### Conflict of interest statement

The authors declare that the research was conducted in the absence of any commercial or financial relationships that could be construed as a potential conflict of interest.

## Supplementary material

Supplemental File 1 **Neighbor-joining bootstrap tree phylogeny based on full-length amino acid sequences of stearoyl-acyl carrier protein-desaturase (SAD) gene families in Criollo and Matina1-6 genotypes of cacao**.

Supplemental File 2 **Melt curve analysis of 10 candidate reference genes in cacao**.

Supplemental File 3 **Melt curve analysis of SAD isoforms in cacao**.
