# Supplementary figures and images for: Characterization of a stearoyl-acyl carrier protein desaturase gene family from chocolate tree, Theobroma cacao L
Source: Front Plant Sci. 2015 Apr 14;6:239. doi: 10.3389/fpls.2015.00239 (PMC4396352; doi:10.3389/fpls.2015.00239)

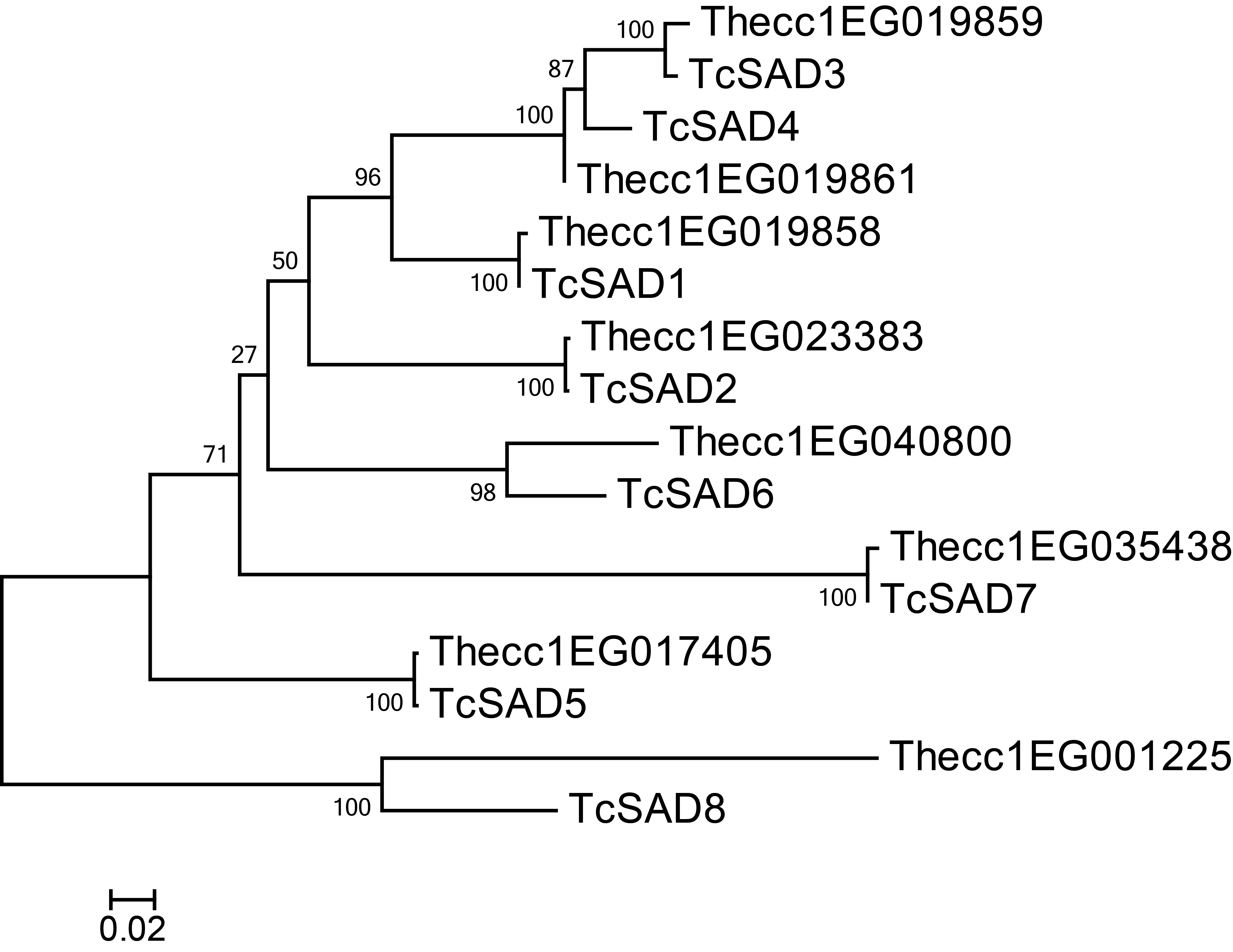

Supplement: Supplemental File 1 — Neighbor-joining bootstrap tree phylogeny based on full-length amino acid sequences of stearoyl-acyl carrier protein-desaturase (SAD) gene families in Criollo and Matina1-6 genotypes of cacao. [file Image1.JPEG]

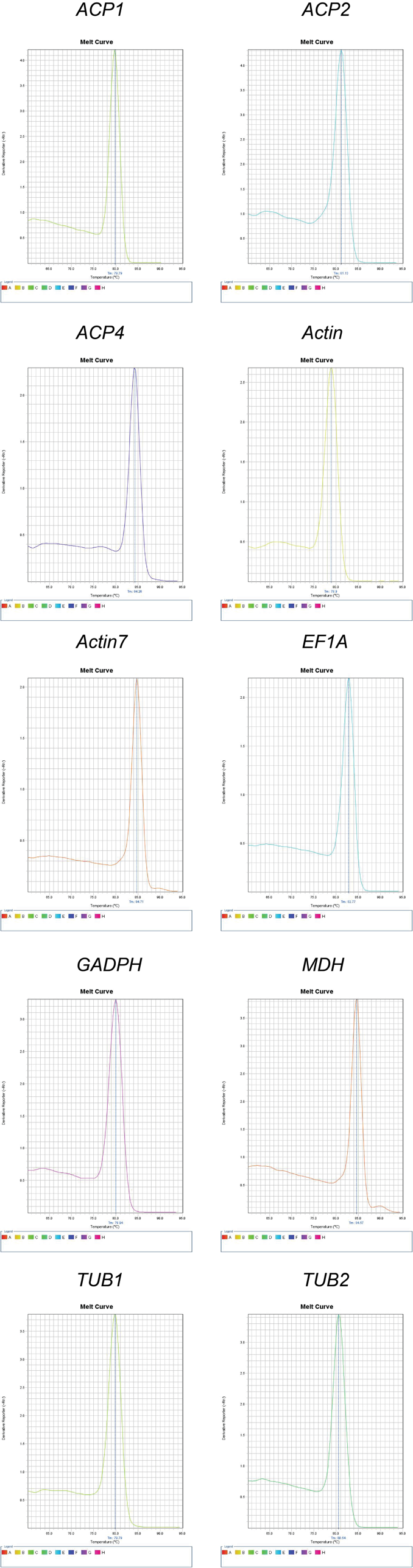

Supplement: Supplemental File 2 — Melt curve analysis of 10 candidate reference genes in cacao. [file Image2.JPEG]
