# Supplementary material for: Characterization of a stearoyl-acyl carrier protein desaturase gene family from chocolate tree, Theobroma cacao L
Source: Front Plant Sci. 2015 Apr 14;6:239. doi: 10.3389/fpls.2015.00239 (PMC4396352; doi:10.3389/fpls.2015.00239)
Supplement: Supplementary file 4 [file Table1.DOCX]

**Supplemental Table 1 Primer sequences used in RT-qPCR for reference gene expression analysis.**

| Gene | Locus ID | Position of primers | Primer sequences | Amplicons size |
| --- | --- | --- | --- | --- |
| ACP1 | Tc01g039970 | 3'UTR | 5'-GGAAAGCAAGGGTGTCTCGTTGAA-3' | 198 bp |
|  |  | 3'UTR | 5'-GCGAGTTGAAATCTGCTGTTGTTTGG-3' |  |
| ACP2 | Tc06g020860 | CDS | 5'-AAGTGCCATCTTGAGGCATGTGAG-3' | 83 bp |
|  |  | CDS | 5'-GCTTGAACATGTTTCCTCCGCAAGTG-3' |  |
| ACP4 | Tc05g024590 | CDS | 5'-GGCATCCATGGCTGGTTCATCAAT-3' | 141 bp |
|  |  | CDS | 5'-CGAGCAGGCATTGGACGCAAATTA-3' |  |
| TUB1 | Tc06g000360 | 3'UTR | 5'-GGAGGAGTCTCTATAAGCTTGCAGTTGG-3' | 77 bp |
|  |  | 3'UTR | 5'-ACATAAGCATAGCCAGCTAGAGCCAG-3' |  |
| TUB2 | Tc08g003640 | 3'UTR | 5'-GCTGAGCATGGCCAAGTTATTGCT-3' | 98 bp |
|  |  | 3'UTR | 5'-ACCTGGAGCTTGGAGGATCGATTT-3' |  |
| Actin | Tc10g003310 | 3'UTR | 5'-TTCTGGTGCAGCTTGGAACTTTGC-3' | 82 bp |
|  |  | 3'UTR | 5'-AGTTCATCGTCACTCCAACATGAGAACA-3' |  |
| Actin7 | Tc01g010900 | 3'UTR | 5'-AGGTGGAGATCATTGAAGGAGGGT-3' | 87 bp |
|  |  | 3'UTR | 5'-ACCAGCGGTCATCACAAGTCACAA-3' |  |
| EF1α | Tc10g005060 | CDS | 5'-CCAAGAAGGGTGGCAAGTGAAACT-3' | 141 bp |
|  |  | 3'UTR | 5'-AAAGATCTCGCCACCGTCTGTCAA-3' |  |
| GAPDH | Tc03g014400 | CDS | 5'-TGCCAAGCTTGTCTCGTGGTATGA-3' | 111 bp |
|  |  | 3'UTR | 5'-CCGAAGCAATACTGCCAAGCAACT-3' |  |
| MDH | Tc04g025530 | 3'UTR | 5'-TGTAACTCACTTTGCCTGTGCCTG-3' | 127 bp |
|  |  | 3'UTR | 5'-CACTACACGTCAGCAACAAGTGATGG-3' |  |
