# Supplementary material for: Characterization of a stearoyl-acyl carrier protein desaturase gene family from chocolate tree, Theobroma cacao L
Source: Front Plant Sci. 2015 Apr 14;6:239. doi: 10.3389/fpls.2015.00239 (PMC4396352; doi:10.3389/fpls.2015.00239)
Supplement: Supplementary file 5 [file Table2.DOCX]

**Supplemental Table 2 Primer sequences used in RT-qPCR for SAD expression pattern analysis.**

| Gene | Position of primers | Primer sequences | Amplicon size |
| --- | --- | --- | --- |
| TcSAD1 | 3'UTR | 5'-GTCACTGTGTGGCAAGTGTCTTCT-3' | 136 bp |
|  | 3'UTR | 5'-CCACACACCTCCCAAACAATTCCT-3' |  |
| TcSAD2 | 3'UTR | 5'-TGAGGAGATGGTTGCAGTAGGTCA-3' | 142 bp |
|  | 3'UTR | 5'-ACGAGACAGGAAACGCTTACCGA-3' |  |
| TcSAD3 | CDS | 5'-AATTAGAAGGCTGGAGGAGAGAGC-3' | 146 bp |
|  | 3'UTR | 5'-GAGAAGACACTTGCAGGGAGAAGA-3' |  |
| TcSAD4 | 3'UTR | 5'-TTTCCTCTCCCTGCAAGTGTCTTC-3' | 82 bp |
|  | 3'UTR | 5'-GGATCTATTCTTTGTGGGTTGACCAG-3' |  |
| TcSAD5 | CDS | 5'-ACAGGTCTTTCTGCCGAGGGTTAT-3' | 136 bp |
|  | CDS | 5'-AAATCCAGCTGAAAGGAGCGCTTG-3' |  |
| TcSAD6 | 5'UTR | 5'-CAAGCGGCCAATTAAAGGTTGAG-3' | 92 bp |
|  | CDS | 5'-TGTTGTGGGATGGAGTGAGT-3' |  |
| TcSAD7 | CDS | 5'-ACGAGCGGGCCAAGAAGAT-3' | 99 bp |
|  | 3'UTR | 5'- AAGCCGCCTTGCCTCCTTCATTT-3' |  |
| TcSAD8 | CDS | 5'-TCCAAAGTCTGCATGGAAGGTGGA-3' | 81 bp |
|  | CDS | 5'-AGGGAATGAGTCCTTCGAAACCCA-3' |  |
